# Expanding the Noonan spectrum/RASopathy NGS panel: Benefits of adding *NF1* and *SPRED1*

**DOI:** 10.1002/mgg3.1180

**Published:** 2020-02-27

**Authors:** Leora Witkowski, Mitchell W. Dillon, Elissa Murphy, Matthew S Lebo, Heather Mason‐Suares

**Affiliations:** ^1^ Departments of Pathology Harvard Medical School and Brigham and Women's Hospital Boston MA USA; ^2^ Laboratory for Molecular Medicine Partners HealthCare Personalized Medicine Cambridge MA USA

**Keywords:** Legius syndrome, Neurofibromatosis type 1, Neurofibromatosis‐Noonan syndrome (NFNS), *NF1*, Noonan syndrome (NS), Noonan syndrome with multiple lentigines (NSML), RASopathy, *SPRED1*, Watson syndrome

## Abstract

**Background:**

RASopathies are a group of disorders caused by disruptions to the RAS‒MAPK pathway. Despite being in the same pathway, Neurofibromatosis Type 1 (NF1) and Legius syndrome (LS) typically present with phenotypes distinct from Noonan spectrum disorders (NSDs). However, some NF1/LS individuals also exhibit NSD phenotypes, often referred to as Neurofibromatosis‐Noonan syndrome (NFNS), and may be mistakenly evaluated for NSDs, delaying diagnosis, and affecting patient management.

**Methods:**

A derivation cohort of 28 patients with a prior negative NSD panel and either NFNS or a suspicion of NSD and café‐au‐lait spots underwent *NF1* and *SPRED1* sequencing. To further determine the utility and burden of adding these genes, a validation cohort of 505 patients with a suspected RASopathy were tested on a 14‐gene RASopathy‐associated panel.

**Results:**

In the derivation cohort, six (21%) patients had disease‐causing *NF1* or *SPRED1* variants. In the validation cohort, 11 (2%) patients had disease‐causing variants and 15 (3%) had variants of uncertain significance in *NF1* or *SPRED1*. Of those with disease‐causing variants, 5/17 only had an NSD diagnosis.

**Conclusions:**

Adding *NF1* and *SPRED1* to RASopathy panels can speed diagnosis and improve patient management, without significantly increasing the burden of inconclusive results.

## Graphical abstract

Adding the NF1 and SPRED1 genes to Noonan spectrum disorder/RASopathy NGS gene panels modestly increases clinical diagnoses without significantly increasing the VUS burden. Since a diagnosis of NF1 or LS would change patient management, the NF1 and SPRED1 genes should be included on all to Noonan spectrum disorder/RASopathy NGS gene panels, including those used prenatally.

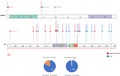

## 1 INTRODUCTION

RASopathies are a group of autosomal dominant disorders caused by pathogenic variants in genes within the RAS‒MAPK pathway. They include the disorders historically referred to as “Noonan spectrum disorders” (NSD: Noonan syndrome (NS; MIM:163950), CBL syndrome (MIM:613563), Noonan syndrome‐like disorder with loose anagen hair (MIM:607721), cardio‐facio‐cutaneous syndrome (CFC; MIM:115150), Costello syndrome (MIM:218040), and Noonan syndrome with multiple lentigines (NSML; MIM:151100)), as well as Neurofibromatosis Type 1 (NF1; MIM:162200) and Legius syndrome (LS; MIM:611431) (Tartaglia & Gelb, 2010). NSDs are caused by gain‐of‐function or altered activity germline variants in the *PTPN11, RAF1, SOS1, RIT1, HRAS, KRAS, NRAS, SHOC2, BRAF, MAP2K1, MAP2K2,* and *CBL*, while NF1 and LS are caused by loss‐of‐function germline variants in the *NF1* and *SPRED1*, respectively (Tartaglia & Gelb, 2010). In addition, there are emerging genes (e.g.*, RASA2, PPP1CB,* and *SOS2)* with limited to strong association to RASopathy‐associated pathognomonic features, as well as *LZTR1*, which is associated with a dominant and recessive form of disease (Grant et al., 2018).

Despite being caused by genes in the same pathway, NF1 and LS typically present distinctly from NSDs. NF1 typically presents with > 6 cafe‐au‐lait spots (CALs), peripheral neurofibromas, Lisch nodules, optic gliomas, skin fold freckling, skeletal dysplasia and absolute macrocephaly, whereas LS typically presents with multiple CALs, relative or absolute macrocephaly, axillary or inguinal freckling, and a lack of Lisch nodules or neurofibromas (Brems et al., 2012; Gutmann et al., 2017; Tartaglia & Gelb, 2010). Furthermore, individuals with LS or NF1 do not typically present with cardiac defects (2% of NF1 affecteds and few reports in LS) or facial dysmorphism (Brems et al., 2012; Lin et al., 2000). In contrast, NSDs typically present with congenital heart defects (mainly pulmonary valve stenosis (PVS) or hypertrophic cardiomyopathy), short stature, dysmorphic facial features (e.g., epicanthal folds, hypertelorism, down‐slanting eyes, short‐broad nose, deep grooved philtrum, small chin, and tall forehead), skeletal deformities (mainly scoliosis or pectus deformities), relative macrocephaly, and may have a small number of CALs (Tartaglia & Gelb, 2010; Williams, Dagli, & Battaglia, 2008).

In rare cases, some individuals with disease‐causing variants in the *NF1* or *SPRED1* may present with multiple NSD pathognomonic features, especially those associated with NS or NSML, and a lack of NF1 features (Tartaglia & Gelb, 2010). For example, Neurofibromatosis‐Noonan syndrome (NFNS) and Watson syndrome (WS), both caused by pathogenic variants in the *NF1*, present with phenotypes similar to NSDs: NFNS presents with a NS facial gestalt, short stature, skeletal defects, and multiple CALs, while WS presents with short stature, PVS, multiple CALs, and intellectual disability (Tartaglia & Gelb, 2010). Furthermore, both NFNS and WS exhibit reduced expressivity of pathognomonic NF1 features (e.g., Lisch nodules, neurofibromatosis, and internal tumors) as compared to classic NF1 (Tartaglia & Gelb, 2010). Accordingly, adult patients with a clinical diagnosis of NS or NSML and disease‐causing variants in the *NF1* gene do not always meet clinical criteria for NF1 (Chen et al., 2014; Croonen, Yntema, van Minkelen, van den Ouweland, & van der Burgt, 2012; Wu et al., 1996).

Many individuals with NFNS may be worked up for an NSD prior to the recognition or development of NF1‐like features, resulting in an incorrect or delayed clinical diagnosis, attributable to the reduced expressivity of pathognomonic NF1 features in NFNS individuals or lack of some NF1 features in children under 6 years of age (Gutmann et al., 2017; Tartaglia & Gelb, 2010). However, early onset of severe and potentially life‐threating NF1 features have been reported, making early screening crucial (Gutmann et al., 2017). The addition of the *NF1* and *SPRED1* genes to Noonan spectrum and RASopathy gene panels should reduce the time to diagnosis for such individuals and allow for appropriate medical management. Hence, many clinical laboratories now offer large RASopathy panels that include the *NF1* and *SPRED1*, and this study quantifies the diagnostic yield of such testing.

## 2 MATERIALS AND METHODS

### 2.1 Ethical Compliance

This study was approved by Partner's Healthcare Institutional Review Board.

### 2.2 Study Population

The derivation cohort included 28 deidentified patients with a suspicion of NSD and cafe‐au‐lait spots or NFNS, who previously tested negative for an NSD gene panel. Their ages ranged from 8 months to 35 years, with a mean age of 6.8 years. Twenty‐one patients were negative for variants in eight RASopathy‐associated genes (*BRAF*, *HRAS*, *KRAS*, *MAP2K1*, *MAP2K2*, *PTPN11*, *RAF1*, and *SOS1*), while seven patients were negative for variants in 10 RASopathy‐associated genes (*CBL* and *NRAS* in addition to the previous eight). The validation cohort included 505 patients with a clinical diagnosis or suspicion of a RASopathy and no prior molecular testing for RASopathies. Their ages ranged from 1 month to 54 years, with a mean age of 9.7 years. The validation cohort was tested using a 14‐gene panel that included the *NF1* and *SPRED1* (see Table S1 for complete gene list).

### 2.3 Sequencing

DNA was extracted from peripheral blood samples using PureGene Blood Core Kit B (Qiagen). For the derivation cohort, 28 DNA samples were tested using next generation sequencing (NGS) of the *NF1* (NM_000267.3) and *SPRED1* (NM_152594.3) genes. For the validation cohort, all 505 samples were tested using next generation sequencing (NGS) of 14 RASopathy‐associated genes (Table S1) as previously described (Pugh et al., 2014). Briefly, NGS was performed by oligonucleotide hybridization‐based DNA capture (SureSelect; Agilent) followed by sequencing using the MiSeq‐M01450 instrument (150‐base paired end mode; Illumina). Sequence reads were aligned to the reference sequence (GRCh37) using bwa‐mem v0.7.10, followed by variant calling using GATK, version 1.0.4705 (McKenna et al., 2010). For the validation cohort, Sanger sequencing was used to confirm all clinically significant variants and fill in regions with insufficient coverage. Methods used for polymerase chain reaction and Sanger sequencing have been previously described (Zimmerman et al., 2010). Copy number variants (CNVs) were identified via an NGS‐based detection tool (VisCap) (Pugh et al., 2016), but were only available for 23 patients in the derivation cohort and 281 patients in the validation cohort due to NGS data quality. Confirmation of CNVs was done using ddPCR as previously described (Ceyhan‐Birsoy et al., 2015).

### 2.4 Variant classification

Variant classification was based on the 2015 guidelines by the American College of Medical Genetics and the Association of Molecular Pathology (Richards et al., 2015). Variants of uncertain significance (VUS) were further subcategorized into three categories: VUS‐favor pathogenic when there is a suspicion of a pathogenicity, VUS‐favor benign when the evidence suggests the variant does not contribute to disease, and VUS when there is a lack of or conflicting evidence. Although clinical features observed in our cohorts were not used to classify the identified variants, physician‐reported clinical findings in patients with pathogenic or likely pathogenic *NF1* or *SPRED1* variants are described in Tables 1 and 2.

**Table 1:** Clinical features of derivation cohort cases with Pathogenic/Likely Pathogenic NF1 or SPRED1 Variants

**ID**	1	2	3	4	5	6
**Age**	5Y	1Y	7Y	2Y	10M	9Y
**Sex**	M	F	M	F	M	M
**Gene**	NF1	NF1	NF1	NF1	NF1	SPRED1
**cDNA change**	c.2033_2034insC	c.3357delA	c.3827G>A	c.5488C>T	c.(?_−50)_(*, *68_?)del	c.1A>G
**Amino acid change**	p.(Ile679Aspfs*, *21)	p.(Val1120Leufs*, *22)	p.(Arg1276Gln)	p.(Arg1830Cys)	Whole gene deletion	p.(Met1?)
**Classification**	Pathogenic	Likely Pathogenic	Pathogenic	Pathogenic	Pathogenic	Likely Pathogenic
**Clinical Diagnosis/Suspicion**	NSML	NFNS or NSML	NS	Unspecified	NFNS	Unspecified
Skin						
Café au lait spots (*n*)	Y (multiple)	Y (multiple)	Y	Y	Skin findings, unspecified	Y (multiple)
Lentigines	N	Y	N	N	N	N
Inguinal/ Axillary freckling	N	Y	N	N	N	Y
Lisch Nodules	N	N	N	N	N	N
Wide‐spaced nipples	N	Y	N	N	N	N
Heart defect						
Pulmonic valve stenosis	N	N	N	Y	Y	Y
Other	HCM	N	N	N	N	N
Facial Dysmorphism						
Epicanthal Folds	N	Y	N	Y	Y	N
Ptosis	N	N	N	N	Y	N
Low Nasal Bridge	N	N	N	N	Y	N
Macrocephaly	N	N	N	N	N	N
Hypertelorism	Y	Y	N	N	Y	Y
Downward eye slant	Y	N	N	N	Y	Y
Low set/ posteriorly rotated ears	N	N	N	N	Y	N
Papillomas	N	N	N	N	N	N
Coarseness	N	N	N	N	N	N
Short/Thick neck	N	N	N	N	N	N
**Short Stature**	N	N	N	N	Y (10%ile)	N
**Neurological Features**						
Developmental Delay	N	Y	N	N	Y	N
Learning Disabilities	N	N	N	N	N	Y
ID	N	N	N	N	N	N
Seizures	N	N	N	N	N	N
Skeletal Features						
Pectus excavatum	N	Y	N	N	N	N
Pectus carinatum	N	N	N	N	N	N
Scoliosis	N	N	N	N	N	N
**Neurofibromas**	N	N	N	N	N	N
**Other**	N	Broad nasal root and tip, prominent forehead, scaphocephaly	Hypospadias	N	N	N
**Family History**	N	N	N	N	N	N

* Transcripts: *NF1* (NM_000267.3) and *SPRED1* (NM_152594.3)

**Table 2:** Clinical features of validation cohort cases with Pathogenic/Likely Pathogenic NF1 or SPRED1 Variants

**ID**	7	8	9	10	11	12	13	14	15	16	17
Age	4M	25Y	18Y	2Y	1Y	4Y	9M	4Y	5.5M	7Y	3Y
Sex	F	F	M	M	F	F	F	M	M	M	F
Gene	NF1	NF1	NF1	NF1	NF1	NF1	NF1	NF1	NF1	NF1	SPRED1
cDNA change	c.204+1G>T	c.2288T>C	c.2970_2972delAAT	c.3827G>A	c.3827G>A	c.3827G>A	c.4330A>G	c.5305C>T	c.6854_6855insA	c.(?_−50)_(*68_?)del	c.423+2T>C
Amino acid change	p.?	p.(Leu763Pro)	p.(Met992del)	p.(Arg1276Gln)	p.(Arg1276Gln)	p.(Arg1276Gln)	p.(Lys1444Glu)	p.(Arg1769*)	p.(Tyr2285*)	Whole gene deletion	p.?
Classification	Pathogenic	Likely Pathogenic	Pathogenic	Pathogenic	Pathogenic	Pathogenic	Pathogenic	Pathogenic	Pathogenic	Pathogenic	Pathogenic
Clinical Diagnosis/Suspicion	NFNS	NS	Legius or NFNS	Unspecified	NS	NS	NFNS or CFC	NF1 + other features	NFNS	NFNS	NF1
Skin											
Café au lait spots (*n*)	Y (multiple)	N	Y (11)	N	Y (7)	N	Y (15)	Y (multiple?)	Y (5)	Y (many)	Y (>6)
Lentigines	N	N	N	N	N	N	N	Y	N	N	N
Inguinal/Axillary freckling	N	N	N	N	N	N	N	Y	N	N	Y
Lisch Nodules	N	N	N	N	N	N	N	N	N	N	N
Wide‐spaced nipples	N	N	N	N	N	Y	N	N	N	N	N
Heart Defect											
Pulmonic valve stenosis	Y	N	N	N	Y	Y	N	N	N	N	N
Other	Heart murmur	MVP	Vasculopathy	N	N	N	PFO	N	N	MVP	N
Facial Dysmorphism											
Epicanthal Folds	N	N	N	N	Y	N	Y	Y	N	Y	N
Ptosis	N	N	N	N	N	Y	N	N	Y	N	N
Low Nasal Bridge	N	N	N	N	Y	Y	Y	N	N	N	wide nasal bridge
Macrocephaly	N	N	N	N	Y	N	Y	Microcephaly	N	Y	N
Hypertelorism	N	N	N	N	Y	Y	Y	N	N	N	N
Downward eye slant	N	N	N	N	N	N	Y	N	Y	N	N
Low set/ posteriorly rotated ears	N	Y	N	N	N	Y	Y	N	Y	N	N
Papillomas	N	N	N	N	N	N	N	N	N	N	N
Coarseness	N	N	N	N	N	N	Y	N	N	Y	Y (prominent lips)
Short/Thick neck	N	Y	N	N	N	Y	N	N	N	N	N
Short Stature	N	Y	Y	N	Y (1%ile)	N	N	N	N	N	Y (3%ile)
Neurological Features											
Developmental Delay	Y	N	N	N	Y	Y	Y	Y	N	N	N
Learning Disabilities	N	N	Y	N	N	Y	N	Y	N	N	N
Intellectual Disability	N	N	N	N	N	Y	N	Y	N	N	Y
Seizures	N	N	Y	N	N	N	N	N	N	N	N
Other	N	N	N	N	N	N	N	N	N	ADHD	N
Skeletal Features											
Pectus excavatum	N	N	N	N	N	Y	Y	N	N	Y	N
Pectus carinatum	N	N	N	N	N	N	N	N	N	N	N
Scoliosis	N	N	Y	N	N	Y	N	N	N	N	N
Neurofibromas	N	N	N	N	N	N	N	N	N	N	N
Other	Plagiocephaly	N	Possible malignancies	Hearing loss	Hepatomegaly	N	Hypotonia, Laryngomalacia	Hemangioma 6mo, encephalopathy	Tall forehead, cryptorchidism	Decreased hair pigmentation at midline frontal area	Bilateral vesicoureteral reflux, Short hands and broad fingers, similar feet
Family History	Mother has hypothyroidism, maternal cousin has HLHS	Patient is 22 weeks pregnant, fetus has cardiomegaly	Father has CALMs	None	None	Mother has learning disabilities, AFIB, and CALs, sister has NF1 and is carrier	Mother has wide‐spaced eyes, tall stature. Mother had 3 SABs.	Multiple maternal relatives have CALs and developmental problems	Maternal uncle died near term, was very small, soft head; additional uncle died at 1 yr	Multiple maternal relatives have 2–3 CALs	None

Abbreviations: CALs, Cafe au lait macules; HLHS, Hypoplastic left heart syndrome; MVP, Mitral Valve Prolapse; PFO, patent foramen ovale; SAB, Spontaneous abortion

* Transcripts: *NF1* (NM_000267.3) and *SPRED1* (NM_152594.3)

## 3 RESULTS

### 3.1 Positive findings

Testing of the *NF1* and *SPRED1* genes in 28 patients in the derivation cohort revealed six (21%) patients with likely pathogenic or pathogenic variants. Proband 6 had a likely pathogenic variant in the *SPRED1*, and Probands 1–5 had a pathogenic or likely pathogenic *NF1* variant: one full gene deletion, two frameshift variants, and two missense variants (Table 1).

Sequencing of all 14 RASopathy‐associated genes in the 505 patients in the validation cohort identified 11 (2%) patients with disease‐causing variants (Table 2) and 15 (3%) patients with a VUS (*n* = 11) or VUS‐likely benign (*n* = 4) (Table S2) in the *NF1* or *SPRED1* genes. One patient (Proband 17) with a clinical suspicion of NF1 had the pathogenic c.4232T>C variant in *SPRED1*. Likely pathogenic or pathogenic variants in *NF1* included one deletion of the entire *NF1* gene, one splice variant, two nonsense variants, one in‐frame deletion, and three missense variants (Table 2). Adding *NF1* and *SPRED1* to the panel increased the diagnostic yield from 23.5% (119/505) to 25.7% (130/505).

### 3.2 Clinical features

The derivation cohort demonstrated that disease‐causing *NF1* or *SPRED1* variants could be associated with a predominance of NSD phenotypes and limited NF1 pathognomonic features. Of the six positive patients in the derivation cohort, one had a clinical suspicion of NFNS, one had a clinical suspicion of NFNS or NSML, one had a clinical diagnosis of NSML, one had a clinical suspicion of NS, and two were unspecified (Table 1). The patients with diagnoses of only NS or NSML did not have any other features of NF1 or LS except CALs. Four of six (66%) patients had NS‐like facial features not typically seen in NF1 or LS. Three patients had pulmonary valve stenosis (PVS) and one patient with a frameshift variant in the *NF1* gene had hypertrophic cardiomyopathy, rare features of NF1 and LS but common in NSDs (Brems et al., 2012; Lin et al., 2000). Only two patients had inguinal and/or axillary freckling, a feature typically seen in NF1 and LS (Brems et al., 2012; Gutmann et al., 2017; Tartaglia & Gelb, 2010); although, one of these two patients was also reported to have lentigines. Distinguishing axillary freckling from multiple lentigines, a cardinal feature of NSML, may be difficult (Carcavilla et al., 2011). None of the patients had Lisch nodules or neurofibromas, which may be due to the young age for some of these patients: 66% under 6 years of age.

There was a lack of pathognomonic NF1 features, and an increase of NSD features, in the 11 positive patients in the validation cohort. Their original clinical diagnoses include five with a clinical suspicion of NFNS, three with a clinical suspicion of NS, one with a suspicion of NF1 with other features, one with a suspicion of an unspecified RASopathy, and the one patient with a pathogenic *SPRED1* variant had a clinical diagnosis of NF1. Of the three patients with only NS indicated, only one had any NF1 features: CALs. Eight patients had NS‐like facial features. Six patients had cardiac abnormalities, three of whom had PVS, which is typically seen in only 1% of NF1 affecteds (Lin et al., 2000). Eight patients had neurological features, including three (none with a full *NF1* deletion) who met criteria for intellectual disability, a rare finding in NF1 or LS without a full *NF1* deletion (Brems et al., 2012; Tartaglia & Gelb, 2010). This cohort ranged from 4 months to 25 years (73% under 6 years of age), which may explain the lack of NF1 features in some of the younger patients.

## 4 DISCUSSION

Diagnosing a specific RASopathy without genetic testing can be difficult, as some patients present with unconventional phenotypes and some pathognomonic features have age‐related penetrance. In this study, no positive patients had neurofibromas or Lisch nodules, pathognomonic NF1 features that typically present before 10 years of age (Gutmann et al., 2017). Many positive patients may have been too young (71% under 6 years of age) to develop all pathognomonic NF1 features and post‐testing phenotypes were often unavailable, as we were limited to clinical information acquired via a pretest questionnaire, which may explain why some patients failed to meet NF1 or LS clinical criteria (Tables 1 and 2). However, two patients (Probands 8 and 14) were 18 years or older and, therefore, should have developed pathognomonic NF1 features. While Proband 8 had a clinical suspicion of NFNS based on multiple CALs, she did not present with any of the pathognomonic NF1 features. The *NF1* p.(Met992del) variant that she carries has been previously associated with learning difficulties and a lack neurofibromas or Lisch nodules (Koczkowska et al., 2019), consistent with her presentation. Proband 14, at the age of 25, had no NF1 phenotypes and was diagnosed with mild NS during evaluation of fetal cardiomegaly in pregnancy. Prenatal testing was not pursued, so it is unknown if the fetal cardiomegaly was associated with the *NF1* variant she carried, although studies have associated prenatal ultrasound findings with NF1 (Carss et al., 2014; Drury et al., 2015; McEwing et al., 2006). The lack of pathognomonic NF1 features seen in these patients and other reported patients (Chen et al., 2014; Croonen et al., 2012; Wu et al., 1996) demonstrate that NF1 can be missed clinically. Alternatively, there have been patients fulfilling NF1 clinical criteria with only disease‐causing *PTPN11* variants (Carcavilla et al., 2011). Therefore, despite the modest increase in molecular diagnosis in our study (increased from 23.5% to 25.7% in the validation cohort with an overall positive rate = 3.2% (17/533) for both cohorts), patients with a suspected RASopathy should be tested on an NGS panel that includes the *NF1* and *SPRED1*.

NFNS syndrome has been associated with an increased rate of missense variants and in‐frame deletions in the GAP domain of the *NF1* gene when compared to the mutation spectrum associated with classic NF1 (Tartaglia & Gelb, 2010). In our cohort, the mutation spectrum included all variant types, with only 5 of 17 (29%) patients having variants in the GAP domain (p.(Arg1276Gln) or p.(Lys1444Glu)). Interestingly, two patients (Patients 5 and 16) had full *NF1* deletions, which is typically associated with a more severe phenotype, including intellectual disability and a high number of cutaneous neurofibromas (Tartaglia & Gelb, 2010). Both patients had neurologic features but neither had neurofibromas, which may be due to their age.

Cost‐benefit considerations support adding the *NF1* and *SPRED1* to the Noonan spectrum disorder/RASopathy NGS gene panels. In our cohort, only 15/505 (3%) patients had a VUS in *NF1* or *SPRED1*, four of which were VUS‐favor benign and not expected to be the cause of disease (Table S2). This rate of VUSs is similar to the VUS rates observed in the other RASopathy genes (Ceyhan‐Birsoy, Miatkowski, Hynes, Funke, & Mason‐Suares, 2018; Leach et al., 2019), suggesting that adding these genes would have a limited burden on the diagnostic laboratory.

## 5 CONCLUSION

Adding the *NF1* and *SPRED1* genes to Noonan spectrum disorder/RASopathy NGS gene panels modestly increases clinical diagnoses without significantly increasing the VUS burden. Since a diagnosis of NF1 or LS would change patient management, *NF1* and *SPRED1* should be included on all Noonan spectrum disorder/RASopathy NGS gene panels.

## CONFLICT OF INTEREST

All authors are employed by a nonprofit clinical genetic testing facility.

## Data Availability

The data that support the findings of this study are openly available in ClinVar at https://www.ncbi.nlm.nih.gov/clinvar/.
