# Supplementary material for: Expanding the Noonan spectrum/RASopathy NGS panel: Benefits of adding NF1 and SPRED1
Source: Mol Genet Genomic Med. 2020 Feb 27;8(4):e1180. doi: 10.1002/mgg3.1180 (PMC7196473; doi:10.1002/mgg3.1180)
Supplement: Supplementary file 1 [file MGG3-8-e1180-s001.pdf]

**Supplementary Table S1. RASopathy Genes on NGS Panel**

| Gene | Transcript |
|---|---|
| *BRAF* | NM_004333.4 |
| *CBL* | NM_005188.3 |
| *HRAS* | NM_005343.2 |
| *KRAS* | NM_004985.3 |
| *MAP2K1* | NM_002755.3 |
| *MAP2K2* | NM_030662.3 |
| *NF1* | NM_001042492.2 |
| *NRAS* | NM_002524.4 |
| *PTPN11* | NM_002834.4 |
| *RAF1* | NM_002880.3 |
| *RIT1* | NM_001256821.1 |
| *SPRED1* | NM_152594.2 |
| *SOS1* | NM_005633.3 |
| *SHOC2** | NM_007373.3 |

***Tested for the only known pathogenic (c.4A>G (p.Ser2Gly)) variant in exon**

**Supplementary Table S2. Variants of Unknown Significance (VUSs) in *NF1* and *SPRED1* Identified on RASopathy Panel**

| Gene | cDNA | Protein | Variant classification (ACMG/AMP Criteria) | Probands |
|---|---|---|---|---|
| *NF1* | c.2410-12T>C | p.(?) | VUS (PM2, PP3) | 1 |
| *NF1* | c.3883A>G | p.(Thr1295Ala) | VUS - favor benign (BP4, PM2, BS2) | 1 † |
| *NF1* | c.4624C>G | p.(Leu1542Val) | VUS (PM2) | 1 |
| *NF1* | c.4835G>T | p.(Arg1612Met) | VUS (PP3, PM2) | 1 |
| *NF1* | c.5481C>G | p.(Ile1827Met) | VUS (PM2) | 1 |
| *NF1* | c.5509G>A | p.(Asp1837Asn) | VUS (PM2_P) | 1 |
| *NF1* | c.6161T>A | p.(Met2054Lys) | VUS (PM2, PP3) | 1 ‡ |
| *NF1* | c.6172A>G | p.(Ile2058Val) | VUS (BS1_P) | 1 |
| *NF1* | c.6617C>T | p.(Thr2206Ile) | VUS - favor benign (PM2, PP3, BS2) | 1† |
| *NF1* | c.7439A>G | p.(His2480Arg) | VUS (PP3) | 1 |
| *NF1* | c.7988C>T | p.(Ser2663Phe) | VUS - favor benign (PM2, PP3, BS2) | 1† |
| *NF1* | c.8162A>G | p.(Gln2721Arg) | VUS (PM2) | 1§ |
| *SPRED1* | c.-12G>T | p.(?) | VUS (PP3, BS1_P) | 1 |
| *SPRED1* | c.424-5A>G | p.(?) | VUS (BP4) | 1 |
| *SPRED1* | c.587C>T | p.(Thr196Ile) | VUS - favor benign (BP4, BS1_P) | 1 |

†Also identified in unaffected parent
‡In homozygous state in proband with a RASopathy and affected sibling
§Also identified in parent, phenotype unknown
